# Tuberculosis and the Tubercle Bacillus

**DOI:** 10.3201/eid1108.050611

**Published:** 2005-08

**Authors:** Jun Liu

**Affiliations:** * University of Toronto, Toronto, Ontario, Canada

**Keywords:** Mycobacterial infections, tuberculosis, bacterial diseases

Mycobacterial infections, including tuberculosis (TB) and leprosy, are bacterial diseases of global importance. An estimated 2 billion people are infected with *Mycobacterium tuberculosis*. Control of TB is complicated by its ease of transmission, difficulty in administering the long-course chemotherapy regimens, and subsequent appearance of multidrug-resistant strains (MDR-TB). This situation is made even worse by the deadly combination of coinfections of HIV and *M. tuberculosis*. New approaches to the control of TB are urgently needed, including development of short-term antimicrobial regimens to minimize the appearance of drug resistance, new drugs to treat MDR-TB patients, and new vaccines with greater efficacy than BCG.

Tuberculosis and the Tubercle Bacillus has many contributors; chapters are provided by experts in many areas of TB research to bring together a comprehensive update of research development in the past decade. The publication of this book is necessary and timely, considering the current urgencies and growing interests of investigators from various fields.

The book is divided into 3 sections, each consisting of multiple chapters on various subjects. The first section focuses on clinical aspects of the disease, including the global impact of TB, clinical and epidemiologic features, as well as diagnosis and treatment. The second section deals with the bacteriology of *M. tuberculosis*, with chapters devoted to molecular genetics, genomics, cell wall structure and synthesis, and metabolism. The third section details the host-pathogen interaction, covering topics such as the intracellular survival of *M. tuberculosis*, host immune response, animal models, and vaccine development.

The book accurately reflects current knowledge of TB and recent research efforts and progresses to the control of the disease. The book flows smoothly from chapter to chapter. Each chapter is clearly written and appropriately referenced. The book focuses primarily on *M. tuberculosis*; research performed on other mycobacterial species is not discussed or only briefly mentioned. Nevertheless, at 584 pages, this book is easily read and is a useful reference for clinicians and basic scientists, including students, laboratory supervisors, and senior scientists.

